# Circulating Inflammatory Mediators as Potential Prognostic Markers of Human Colorectal Cancer

**DOI:** 10.1371/journal.pone.0148186

**Published:** 2016-02-09

**Authors:** Giuseppe Di Caro, Michele Carvello, Samantha Pesce, Marco Erreni, Federica Marchesi, Jelena Todoric, Matteo Sacchi, Marco Montorsi, Paola Allavena, Antonino Spinelli

**Affiliations:** 1 Department of Immunology and Inflammation, Humanitas Clinical and Research Center, Rozzano, Milan, Italy; 2 Department of Colon and Rectal Surgery, Humanitas Clinical and Research Center, Rozzano, Milan, Italy; 3 Laboratory of Gene Regulation and Signal Transduction, Departments of Pharmacology and Pathology, School of Medicine, University of California San Diego, La Jolla, California, United States of America; Shiga University of Medical science, JAPAN

## Abstract

**Background:**

Cytokines and chemokines in the tumor microenvironment drive metastatic development and their serum levels might mirror the ongoing inflammatory reaction at the tumor site. Novel highly sensitive tools are needed to identify colorectal cancer patients at high risk of recurrence that should be more closely monitored during post-surgical follow up. Here we study whether circulating inflammatory markers might be used to predict recurrence in CRC patients.

**Methods:**

Circulating levels of the inflammatory cytokines IL-1, IL-6, IL-10, TNFalpha, CCL2, CXCL8, VEGF and the acute phase protein Pentraxin-3 were measured by ELISA in preoperative serum samples prospectively collected from a cohort of sixty-nine patients undergoing surgical resection for stage 0–IV CRC and associated with post-operative disease recurrence.

**Results:**

Cox multivariate analysis showed that combined high levels (≥ROC cut off-value) of CXCL8, VEGF and Pentraxin3 were associated with increased risk of disease recurrence [HR: 14.28; 95%CI: (3.13–65.1)] independently of TNM staging. Kaplan-Meier analysis showed that CXCL8, VEGF and Pentraxin3 levels were significantly associated with worse survival (P<0.001).

**Conclusions:**

Circulating inflammatory mediators efficiently predicted postoperative recurrence after CRC surgery. Therefore, this study suggest that their validation in large-scale clinical trials may help in tailoring CRC post-surgical management.

## Introduction

Colorectal cancer (CRC) is a leading cause of cancer death in developed countries [1]. Despite advances in diagnostic tests and surgical procedures that usually have curative intent, about half of patients undergoing surgery for CRC with curative intent will experience metachronous metastasis and consequent death [2]. To date pathological Nodal Metastasis Tumor System (TNM) staging remains the benchmark for the prediction of CRC patients who will experience disease progression, despite the core of the original Dukes system [3] is substantially unchanged. In fact, histopathological staging lacks accuracy and an important percentage of patients at greatest risk for disease recurrence are still not detected with this methodology [2] [4]. Novel prognostic biomarkers of CRC would allow identification of subgroup of patients with high risk of tumor relapse that should be considered for frequent surveillance, diagnostic screening and therapeutic intervention.

It has been well established that inflammatory cells in the tumor microenvironment release inflammatory mediators that in turn activate local immune networks to promote development and growth of malignant cells by increasing their proliferation and survival as well as angiogenesis [5] [6] [7–9]. Besides, inflammatory mediators, i.e. chemokines together with angiogenic factors are a trigger for the development of invasive abilities as they increase tumor cells motility and migration that ultimately results in metastatic occurrence [8]. The interconnection between inflammation and angiogenesis is explained by the ability of VEGF and chemokine such as CCL2 and CXCL8 to recruit leukocytes at the tumor site that in turn produce VEGF and inflammatory mediators and thus amplify tumorigenic signal via an autocrine and paracrine loop [8]. Chemokines and cytokines are extensively produced in the tumor microenvironment regardless of the initial triggers [8] and mediators such as IL-1b, TNFa and IL-6 directly promoted the tumor progression in experimental models of colorectal cancer [8, 10].

Inflammatory mediators detected in the serum of patients might mirror the ongoing inflammatory reaction at the tumor site. Consistently, population-based studies showed that individuals with high circulating levels of inflammatory mediators are at greater risk for developing CRC [11] [12, 13]. Thus, serum levels of these markers have the potential to become a paradigm as non-invasive diagnostic tests for the detection of CRC patients [14]. However, few studies on this issue assessed the ability of circulating inflammatory mediators to identify patients who will develop postsurgical recurrences and metachronous metastasis with respect to current clinical prognostic tools for CRC [15–17]. Therefore, well designed prospective studies are needed to better establish their clinical relevance.

In this study, we prospectively sampled preoperative plasma of CRC patients undergoing surgical treatment at our Institution and we assessed whether the levels of cytokines and chemokines represented by IL-1, IL-6, IL-10, TNFalpha, CCL2, CXCL8, VEGF and the acute phase protein Pentraxin-3 might be associated with postoperative disease recurrence in a 5-year follow-up study.

## Material and Methods

### Patient’s enrollment and study design

69 patients who were diagnosed with colorectal tumor, and consecutively underwent radical surgery in Humanitas Research hospital with curative intent between April 2008 and August 2009 were included in this study. After patient gave informed consent to the investigation, a blood sample from each patient was withdrawn at the time of hospital admission and before surgery to be processed for plasma separation. Investigators who were blinded to the results of the levels of immune parameters assembled a clinical database by collecting patients’ demographics, clinical and histopathological data that were recorded on the Institutional’s Intranet server. Tumor clinical and histopathologic staging at diagnosis was determined in all patients by combining histopathologic findings with surgical records and perioperative imaging. Tumor staging was categorized according to the American Joint Committee on Cancer AJCC guidelines. All the patient’s demographics, clinical and histopathological variables were tested for covariance with levels of immune mediators (Table 1). These variables, together with the amount of IL-1, IL-6, IL-10, TNFalpha, CCL2, CXCL8, VEGF and of the acute phase protein Pentraxin-3, were tested as predictors of patient’s outcome, to evaluate the likelihood of tumor relapses with respect to the extent of blood levels of immune mediators. We defined the outcome of CRC patients as a variable including any event (n = 9) of local tumor recurrences (n = 1) or any metachronous distant-organ metastases (n = 8), which is named disease free survival (DFS). The survival of patients was calculated as disease specific survival (DSS) and concerned any death related only to CRC disease (n = 5). To detect or exclude any postsurgical tumor recurrences, patients underwent thoraco-abdominal computed-tomography, abdominal ultrasonography, and chest radiography that were done according to common protocols for surveillance. The observation period started immediately after the surgical procedure and the detection of tumor recurrence or tumor related death was computed from diagnosis until data were censored. The mean follow-up period of the cohort studied was 56.33 months (SD = 15.74 months). If the death event was unrelated to CRC disease, time to follow-up was stopped at the time of patient’s death which was not considered an event of outcome. Postsurgical tumor surveillance follow up data have not been available in 6 out of 69 patients (8.7%) enrolled in the study and were therefore excluded from the outcome analysis according to previous guidelines [18]. Ethics Committee of the Humanitas Clinical and Research Center approved the study, and the referring physician obtained written informed consent from each patient prior to surgery.

**Table 1:** IL-1, IL-6, CXCL8, IL10, CCL2, PTX-3, TNF-a and VEGF values in 69 stage 0-IV CRC patients according to demographics and histopathological features.

		Median values							
	N (%)	IL-1	IL-6	CXCL8	IL10	CCL2	PTX-3	TNF-a	VEGF
**All cases**	69 (100)								
**Age**									
< 70 yrs.	26	0.52	4.69	7.19	5.77	38.41	5.69	13.04	37.59
≥ 70 yrs.	43	0.40	5.86	6.64	5.60	38.81	7.00	10.89	31.92
**Gender**									
Male	35	0.47	6.60	6.98	5.19	40.08	5.76	10.34	37.82
Female	34	0.48	4.95	6.49	6.00	37.12	6.40	11.69	29.33
**TNM staging**									
0-I-II	40	0.44	5.25	5.92	5.31	40.52	5.95	11.17	29.33
III	24	0.40	5.71	6.82	5.84	38.23	6.92	10.47	39.15
IV	4	1.32^a^	12.81	11.48	30.83	40.99	5.02	18.20	106.17^a^
**Tumor site**									
Colon	56	0.46	5.57	6.66	5.84	38.36	6.33	10.98	34.67
Rectum	13	0.54	5.86	6.79	4.14	42.13	5.13	11.54	30.78
**CEA**									
<5ng/ml	42	0.32	4.24	6.79	5.08	38.31	6.20	9.93	31.97
≥5ng/ml	14	1.18 ^a^	9.16	10.57	6.35	43.20	8.37	16.14 ^a^	54.48

Mann Whitney analysis, IL-1, IL-6, CXCL8, IL10, CCL2, PTX-3, TNF-a and VEGF values were entered as dependent continuous variables.

^a^ P<0.05

### Assays

Plasma samples were centrifuged at 3000 rpm for 10 minutes and then kept at -80°C until ELISA assessment. Plasma levels of IL-1, IL-6, IL-10, TNFalpha, CCL2, CXCL8, VEGF were determined by commercially available sandwich enzyme-linked immunosorbent assay (ELISA) kit according to the manufacturer’s instructions. PTX3 in plasma was assayed with an “in house” sandwich ELISA based on the monoclonal antibody MNB4 (capture antibody) and rabbit anti-serum (detection antibody) as previously described [19]. The detection limit is 100 pg/ml and interassay variability is 8%-10%. Normal values of PTX3 are <2 ng/mL. The immune mediators levels in the patients plasma samples were determined by correlating each value duplicates to a standard curve based on a 2-fold serial dilution of recombinant IL-1, IL-6, IL-10, TNFalpha, CCL2, CXCL8, VEGF and PTX3 with known concentration.

### Statistical analysis

Statistical analysis was performed using Epi Info (Version 3.4.3), StatsDirect Statistical software (Version 2.5) and GraphPad Prism software (Version 4.1). The association between immune mediator values and patient’s baseline demographics, clinical and histopathological characteristics and tumor features were estimated by linear regression analysis. To best determine the association of each immune mediator and patient’s prognosis, a receiver operational curve (ROC) analysis was performed. The ROC cut-off value of each continuous distribution was defined to predict the occurrence of patient’s relapses. A Cox proportional hazard model was developed to assess the ROC cut off value of IL-1, IL-6, IL-10, TNFalpha, CCL2, CXCL8, VEGF and PTX3 and other demographic, clinical and histopathological features, in predicting the occurrence of DFS. To assess for confounders, Cox multivariate analysis was performed by entering all the variables with a P value less than 0.20 at univariate analysis. By a backward stepwise elimination approach, non-significant variables and their non-significant interactions, were removed from the model. Kaplan-Meyer curves of DSS were plotted, while log-rank test was used to compare the curves of each subgroup of CRC patients. For each test only two-sided P values lower than 0.05 were considered statistically significant.

## Results

Demographics and histopathological features of CRC patients included in this study and their correlation with quantitative assessment of the preoperative plasma values of immune mediators are shown in Table 1. Subgroup analysis revealed that among all the immune mediators only IL-1b and VEGF values slightly increased in advanced TNM staging (P = 0.06 and P = 0.04, respectively) (Table 1). Interestingly, only IL-1b and TNFa values increased among patients with high CEA blood levels (≥5ng/ml) at the cut-off generally employed for this biomarker, while other immune mediators were not associated with CEA levels (Table 1). Next, we tested the linear correlation coefficients among continuous plasma values of inflammatory markers as shown in Table 2. Linear regression analysis revealed that IL-1b correlated at a great extent with TNF-a (r = 0.94, P<0.001) independently of other immune mediators (Table 2). In addition, CXCL8 values independently linearly correlated with those of IL-6, CCL2 and VEGF (r = 0.34, P = 0.006; r = 0.31 P = 0.01; r = 0.36 P = 0.003, respectively), while IL-10 values linearly correlated only with those of VEGF (r = 0.39, P<0.001) independently of the other immune markers (Table 1). Interestingly, the acute phase protein PTX3 levels did not correlate with any of the immune markers studied (Table 1), suggesting that PTX3 might follow different pathways of activation in CRC patients compared to other immune mediators.

**Table 2:** Multiple regression analysis of IL-1, IL-6, CXCL8, IL10, CCL2, PTX-3, TNF-a and VEGF values in 69 stage 0-IV CRC patients.

Cytokines	IL-1 ^a^	IL-6 ^a^	CXCL8 ^a^	IL-10 ^a^	CCL2 ^a^	PTX-3 ^a^	TNF-a ^a^	VEGF ^a^
IL-1								
r^b^	-							
P	-							
IL-6								
r^b^	0.15	-						
P	0.23	-						
CXCL8								
r^b^	0.03	0.34	-					
P	0.79	**0.006**	-					
IL-10								
r^b^	-0.01	0.06	-0.11	-				
P	0.89	0.61	0.36	-				
CCL2								
r^b^	-0.14	0.18	0.31	-0.01	-			
P	0.25	0.14	**0.01**	0.99	-			
PTX-3								
r^b^	0.10	0.19	0.11	0.06	-0.06	-		
P	0.41	0.13	0.36	0.60	0.63	-		
TNF-a								
r^b^	0.94	-0.05	-0.11	0.07	0.22	-0.12	-	
P	**<0.001**	0.68	0.38	0.57	0.08	0.33	-	
VEGF								
r^b^	0.11	-0.02	0.36	0.39	-0.05	-0.03	-0.10	-
P	0.36	0.88	**0.003**	**0.001**	0.64	0.81	0.43	-

^a^ Multiple linear regression analysis model. Continuous values of IL-1, IL-6, CXCL8, IL10, CCL2, PTX-3, TNF-a and VEGF were entered as dependent continuous variables.

^b^ r = Correlation coefficient.

**Fig 1:**
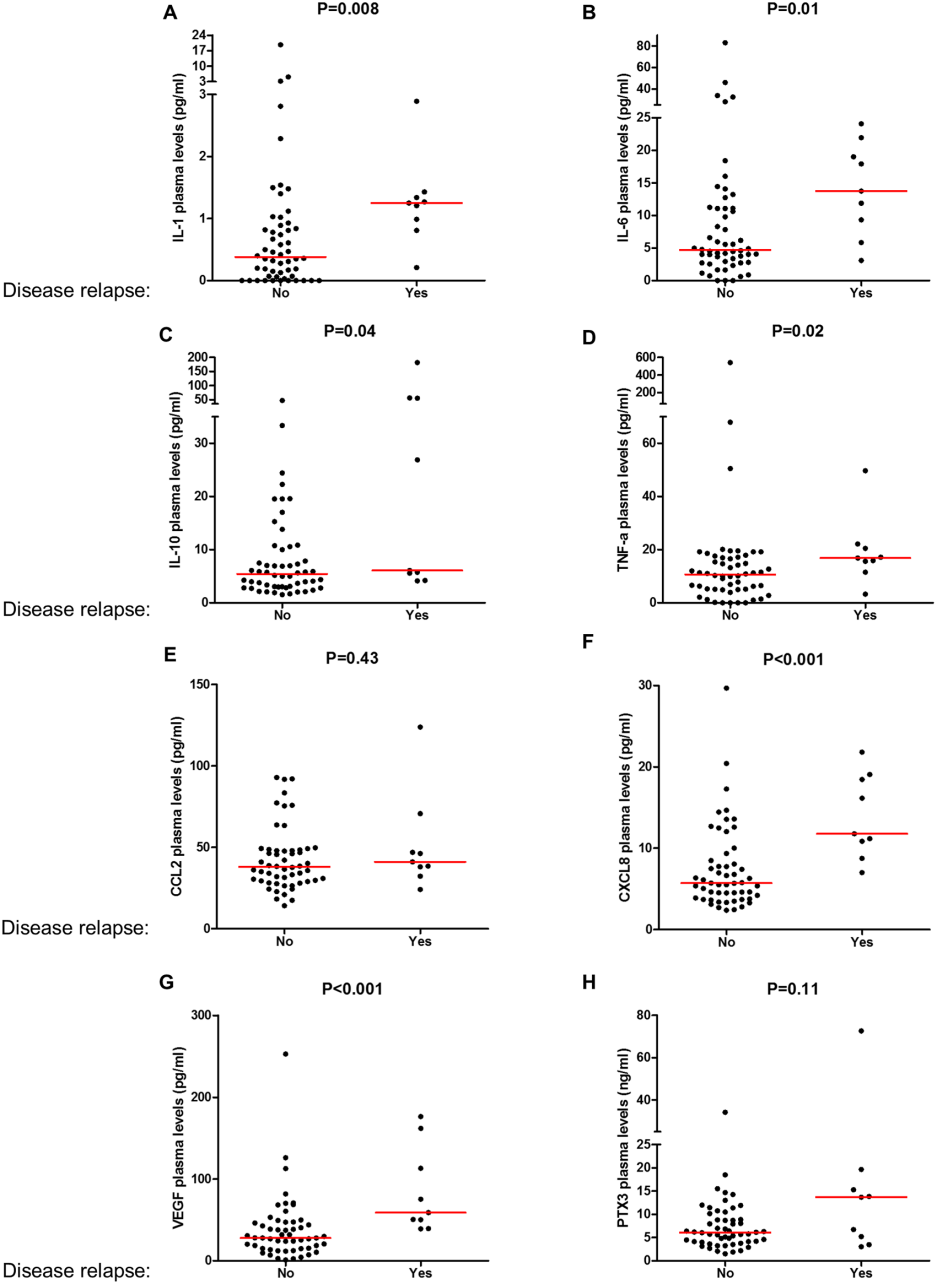
Continuous values of IL-1, IL-6, IL-10, TNF-a, CCL2, CXCL8, VEGF and the acute phase protein Pentraxin-3 in the plasma of colorectal cancers according to the occurrence of postsurgical relapse. The amount of IL-1 (A), IL-6 (B), IL-10 (C), TNF-a (D), IL-8 (F), VEGF (G) significantly increased in patients who experienced disease relapse, while the acute phase protein Pentraxin-3 (H) showed a tendency to associate with worse prognosis. CCL2 (E) levels were not associated with disease relapse.

Subsequently, we investigated the connection between baseline (preoperative) plasma levels of immune mediators and the risk to develop postsurgical CRC recurrences. Continuous distributions of patient’s plasma levels of circulating inflammatory mediators according to postsurgical CRC disease recurrence are shown in Fig 1. We found that patients who did not relapsed (n = 54) had significantly lower plasma concentrations of IL-1 (P = 0.008), IL-6 (P = 0.01), IL-10 (P = 0.04), CXCL8 (P≤0.001), VEGF (P≤0.001) and TNFalpha (P = 0.02) compared to those who experienced recurrence of CRC disease (n = 9) (Fig 1). In contrast, CCL2 values did not change according to the prognosis of CRC patients and the acute phase protein Pentraxin-3 had only a tendency to increase (P = 0.11) among those patients who experienced postsurgical disease recurrence (Fig 1).

Consistent with prior studies [20], to obtain a reproducible and reliable method to benchmark the prognostic values of biomarkers, we calculated ROC curves for each immune markers for CRC patients who had good prognosis (n = 54) versus those who experienced recurrence of disease (n = 9) at follow up (S1 Fig). ROC plot model analysis revealed inflammatory mediators with better sensitivity than specificity such as IL-1b (0.88 and 0.68), IL-6 (0.88 and 0.59), IL-10 (1 and 0.40), TNF-a (1 and 0.05), CCL2 (0.77 and 0.5), CXCL8 (1 and 0.64) and VEGF: (1 and 0.66) at the optimum cutoffs identified by the model (S1 Fig), while PTX-3 had higher specificity that sensitivity (0.55 and 0.90) (S1 Fig). To improve our understanding of the prognostic relevance of immune mediators, we designed a Cox univariate prognostic model (Table 2) based on the threshold values identified by the ROC curve for each inflammatory marker. In this model, CRC patients who had higher plasma levels (≥ROC value) of IL-1 (P = 0.01), CXCL8 (P<0.001), IL-10 (P = 0.01), PTX3 (P = 0.002), IL-6 (P = 0.06), and VEGF (P<0.001) associated with worse prognosis (Table 3), while higher levels of CCL2 (P = 0.31) and TNF-a (P = 0.89) (Table 3) did not. Further, we tested for the independency of these biomarkers. To avoid overfitting of the COX model [21, 22] because of the relatively low amount of events (n = 9) in our cohort, each inflammatory mediator was separately entered in a multivariate model including TNM stage or one other inflammatory mediator (data not shown). In these models, we found that among all inflammatory mediators that were significant at univariate analysis only CXCL8, CCL2 and VEGF values were independently and significantly associated with prognosis (data not shown). In detail, high levels of CXCL8, VEGF and PTX-3 (≥ROC value) identified subgroups of CRC patients [44.4% (28/63); 42.8% (27/63); 15.8% (10/63), respectively] that efficiently detected disease recurrences [47.3% (9/19); 50.0% (9/18); 50.0% (5/5)]. Thus, according to these results we combined the levels of CXCL8, VEGF and PTX-3 (≥ROC value of combined markers) (Table 4) and to adjust for confounders and to test its prognostic independency, we performed a Cox multivariate analysis, with respect to demographics, clinical and histopathologic features (Table 4). Cox multivariate analysis showed that higher levels of combined CXCL8, VEGF and PTX-3 (≥ROC values) associated with worse prognosis [HR: 14.28, 95%CI (3.13–65.1), P<0.001] independently of TNM staging and other variables included in this model (Table 4). Combined high cut-off of CXCL8, VEGF and PTX-3 values was present only in 11% (7/63) of CRC patients, but this subgroup efficiently identified 71.4% (5/7) of relapsing cases. Conversely, a low cut-off of combined inflammatory values of CXCL8, VEGF and PTX-3 identified a 38% (24/63) of CRC patients with 0% (0/24) of relapsing cases. Overall, the predictive value to detect disease recurrences of the combined score of CXCL8, VEGF and PTX-3 was better compared to each of their separate values (all P≤0.05).

**Table 3:** Cytokines as predictors of post-operative disease recurrence in 63 patients with stage 0-IV CRC.

	Tumor Recurrence			COX Analysis	
	no	yes	%	H.R. (95%C.I.)	P
**All cases (N)**	54	9	14.3		
Cytokines values*					
**IL-1**					
Low	37	1	2.6	1.00 ref.	
High	17	8	32.0	11.84 (1.47–95.39)	**0.01**
**IL-6**					
Low	32	1	3.0	1.00 ref.	
High	22	8	26.7	7.20 (0.87–59.24)	0.06
**CXCL8**					
Low	35	0	0	1.00 ref.	
High	19	9	32.1	N.A	**<0.001**
**IL-10**					
Low	22	0	0	1.00 ref.	
High	32	9	22.0	NA	**0.01**
**CCL2**					
Low	27	2	6.9	1.00 ref	
High	27	7	20.6	2.27 (0.45–11.41)	0.31
**PTX3**					
Low	49	4	7.5	1.00 ref	
High	5	5	50.0	9.64 (2.24–41.42)	**0.002**
**TNF-a**					
Low	51	8	13.6	1.00 ref	
High	3	1	25.0	1.15 (0.13–10.13)	0.89
**VEGF**					
Low	36	0	0	1.00 ref	
High	18	9	33.3	NA	<0.001

*Low and high cutoff values for each marker defined by ROC curve analysis.

NA = not available.

**Table 4:** Predictors of post-operative disease recurrence in 63 patients with stage 0-IV CRC.

	Tumor Recurrence		COX Univariate analysis ^b^		COX Multivariate analysis ^a^^,^ ^b^	
	No	Yes	H.R. (95%C.I.)	P	H.R. (95%C.I.)	P
	(n = 54)	(n = 9)				
Cytokine Score*						
Low	24	0				
Medium	28	4	11.51 (3.02–43.83)	<0.001	16.21 (3.56–73.84)	<0.001
High	2	5				
**Age**						
< 70 yrs.	23	2				
≥ 70 yrs.	31	7	2.67 (0.55–12.96)	0.22		
**Gender**						
Male	26	7				
Female	28	2	0.37 (0.08–1.86)	0.23		
**TNM Staging**						
0-I-II	34	2				
III	18	4	3.06 (1.24–7.57)	0.01	3.68 (1.21–11.20)	0.02
IV	1	3				
NA	1	0				
**CEA**						
<5ng/ml	33	5				
≥5ng/ml	8	4	1.47 (0.30–7.33)	0.64		
NA	13	0				
**Tumor Site**						
Colon	44	7				
Rectum	10	2	2.18 (0.57–8.31)	0.26		

* Low and high cutoff values for each marker defined by ROC curve analysis. Low cytokines score was defined by combined lowIL-8-lowVEGF-lowPTX3 values. High score was defined by combined highIL-8-highVEGF-highPTX3 values. Intermediate score was defined by any other values.

^a^ Multivariate COX regression analysis.

^b^ Cytokine score, TNM staging, age and gender were included in the COX multivariate model. By a backward stepwise elimination approach, non-significant variables and their non-significant interactions were removed from the model.

**Fig 2:**
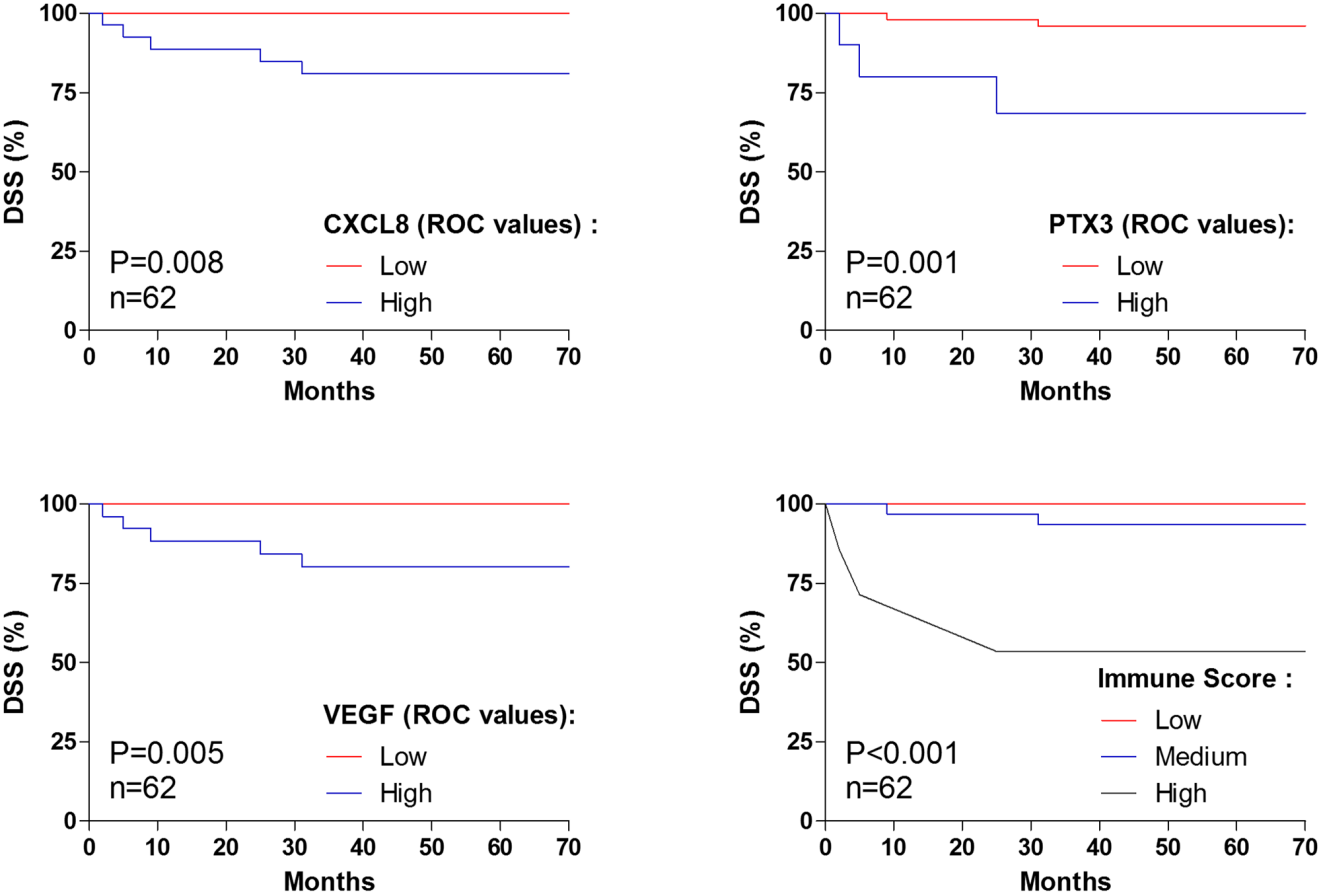
Kaplan—Meier curves showing disease-specific survival (DSS) after resection with curative intent for colorectal cancer according to plasma values of IL-8, PTX-3, VEGF and the immune score. The ROC threshold values of IL-8, PTX3 and VEGF were calculated in the whole cohort (6.79 pg/ml, 31.97pg/ml, 6.29pg/ml, respectively) and were used as a cutoff to select high and low IL-8, PTX-3 and VEGF in colorectal cancer patients. Low immune score was defined by combined lowIL-8-lowVEGF-lowPTX3 values. High immune score was defined by combined highIL-8-highVEGF-highPTX3 values. Intermediate score was defined by any other value. Values above the ROC cutoff were associated with worse patient’s survival for CXCL8 (P = 0.008), PTX3 (P = 0.001), VEGF (P = 0.005) and the immune score (P<0.001), compared to those below ROC cutoff.

Fig 2 shows the DSS curves of all investigated patients sub grouped by the plasma values of CXCL8, VEGF, PTX-3 (≥ROC values) and the immune score. We found an association with worse survival among CRC patients with values of CXCL8 (P = 0.008), VEGF (P = 0.001), PTX-3 (P = 0.005) above the ROC threshold (≥ROC values). The immune score represented by combined ROC values of CXCL8, VEGF and PTX-3 (low, medium, high cutoff) had the ability to predict significantly worse survival (P<0.001) with a better efficiency compared to single immune mediators.

## Discussion

In this study, we showed that inflammatory mediators in the blood of CRC patients collected prior to surgical resection might be candidate biomarkers to predict postsurgical progression of CRC. By characterizing an array of different cytokines we had the possibility to benchmark their prognostic relevance. We showed that CXCL-8, VEGF and the long Pentraxin 3 were the best predictors of prognosis in our CRC study. In addition, a cytokine score based on the combination of the levels of these immune mediators was independent of the conventional TNM staging in identifying post-operative disease recurrences.

The present study followed the REMARK guidelines for the assessment of prognostic tumor markers [23]. However, we acknowledge some weaknesses with the study design. First, we quantified levels of inflammatory mediators before surgery but not during follow up. However, previous studies showed that the levels of a common and unspecific systemic inflammatory marker as CRP did not change in individuals along the years [24, 25]. In addition, we quantified baseline levels of inflammatory mediators the day before surgery and for this reason the type of surgical treatment does not represent a confounding variable as it cannot affect inflammatory mediator levels in our study. Second, we did not examine matched control healthy blood donor since this was beyond the goal of this study. Third, we acknowledge the limited amount of CRC patients included in our study might create overfitting prognostic models. However, the number of events required to test independency of biomarkers is still debated [22] and depends on the extent of the prognostic effect. The limited patient’s number of our cohort rely on its prospective design and the short time span of patient’s enrollment, which decrease selection biases and methodological inconsistencies usually associated with large retrospective cohorts [26]. In addition, we employed ROC curve models to generate reproducible data by measuring the specificity and sensitivity that are useful to benchmark the diagnostic ability of biomarkers across studies [14].

To our knowledge, this is the first study to address the postsurgical prognostic relevance of Pentraxin 3 in CRC. PTX3 is an immune mediator that belongs to the highly conserved family of pentraxins, as well as C reactive protein (CRP) [27]. It has an important role in homeostatic processes such as tissue remodeling, angiogenesis and inflammatory responses ([28], [27], [29] and has been linked to the cancer-related inflammation [30]. In previous clinical studies, PTX3 was found to be increased in other tumors such as liposarcomas [31], glioma [32], lung [20] and prostate cancer [33] compared to paired healthy controls [20, 33] or to benign hyperplasia [33]. However, none of these studies linked PTX3 levels with postsurgical tumor relapses. In addition, our array of cytokines was useful to investigate whether immune mediators correlate with each other in the plasma of human CRC patients. The absence of covariance between Pentraxin-3 and other inflammatory mediators explain its independent impact on prognosis, and further suggests that the combined inflammatory score might increase the detection yield of postsurgical recurrences. Despite strong correlation between TNF-a and IL-1b, these mediators associated with patient’s prognosis to a lesser extent than PTX3 levels, which suggests that these cytokines might have a lower level of involvement in the metastatic relapse of CRC comparing to PTX3. In other studies, PTX3 was shown to be highly induced by LPS/TLRs [34, 35] which can also induce the expression of CXCL8. In this context, it is important to acknowledge that higher PTX3 and CXCL8 blood levels in patients with recurrence of disease in this study represent a mere phenomenological evidence of the inflammatory reaction at the tumor site. Whether these mediators support the local metastatic process and their targeting would affect CRC growth was beyond the aim of this study. Therefore, our data showing the upregulation of serum PTX3 in metastatic CRCs patients is not conflicting with previous mechanistic reports [36] and indeed provide further evidence that this mediator somehow is involved in the tumorigenic reaction.

Together with VEGF, which inhibitor is currently used as an antiangiogenic factor for the treatment of stage IV CRC, CXCL8 is also a potent promoter of angiogenesis that can promote vascularization at the tumor site, proliferation and metastasis of cancer cells and mediate recruitment of tumor infiltrating leukocytes [37–39]. Additionally, our data confirm previous findings on the prognostic abilities of circulating levels of VEGF [40, 41] and CXCL8 [42] in CRC patients demonstrating that this type of methodology is highly reproducible.

Our data suggest that the introduction of circulating inflammatory mediators for detection of post-surgical CRC progression would effectively identify individuals at high risk of recurrence independently of conventional risk predictors. CRC patients identified by high levels of inflammatory mediator plasma levels should be monitored more closely in the follow up regardless of their TNM status which would provide an additional tool in the decision-making process for the postsurgical screening. To conclude, PTX3 and CXCL8 plasma quantification could be coupled with VEGF levels for getting the best yield of postsurgical recurrence detection from the blood samples. These findings, provide the rationale to design large scale clinical studies in order to confirm the prognostic ability of these inflammatory mediators.

## Supporting Information

S1 Fig ROC-plot analysis curves comparing patients according to their postsurgical relapse at the optimum cutoffs identified by the ROC model for all soluble immune mediators studied. ROC plot curves at optimum cutoffs for each immune mediator were calculated and tested for their ability to detect postsurgical tumor recurrences according to their sensitivity and specificity. Sensitivity and Specificity: IL-1 (0,88 and 0,68); IL-6 (0,88 and 0,59); IL-10 (1 and 0,40); TNF-a (1 and 0,05); CCL2 (0,77 and 0,5); CXCL8 (1 and 0,64); VEGF: (1 and 0,66); PTX-3: (0,55 and 0,90). ROC cut-off values: IL-1 (0,81 pg/ml), IL-6 (5,86 pg/ml), IL-10 (4,14 pg/ml), TNFa (49,72 pg/ml), CCL2 (38,03 pg/ml), CXCL8 (7,01 pg/ml), VEGF (39,03 pg/ml), PTX3 (13,68 pg/ml).
(PDF)
