# Correction: Circulating Inflammatory Mediators as Potential Prognostic Markers of Human Colorectal Cancer

**DOI:** 10.1371/journal.pone.0156669

**Published:** 2016-05-26

**Authors:** Giuseppe Di Caro, Michele Carvello, Samantha Pesce, Marco Erreni, Federica Marchesi, Jelena Todoric, Matteo Sacchi, Marco Montorsi, Paola Allavena, Antonino Spinelli

There is an error in the Funding section. The first sentence should read: The work was supported by 16230 Associazione Italiana per la ricerca sul cancro, Marie Curie Actions European Union to GDC.
