# Supplementary figures and images for: Circulating Inflammatory Mediators as Potential Prognostic Markers of Human Colorectal Cancer
Source: PLoS One. 2016 Feb 9;11(2):e0148186. doi: 10.1371/journal.pone.0148186 (PMC4747470; doi:10.1371/journal.pone.0148186)

# ROC plot curves

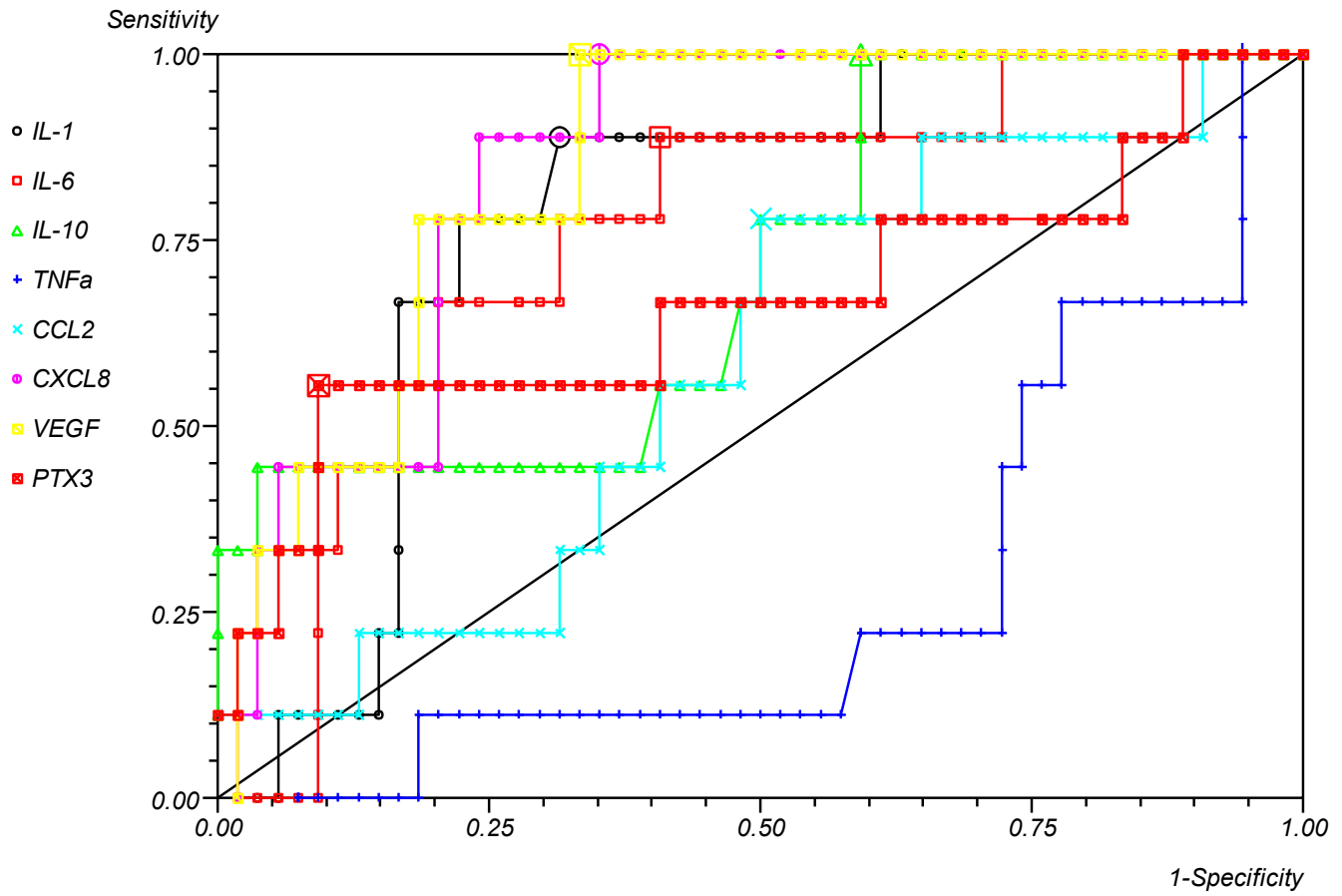

Figure S1.

Supplement: S1 Fig — ROC plot curves at optimum cutoffs for each immune mediator were calculated and tested for their ability to detect postsurgical tumor recurrences according to their sensitivity and specificity. Sensitivity and Specificity: IL-1 (0,88 and 0,68); IL-6 (0,88 and 0,59); IL-10 (1 and 0,40); TNF-a (1 and 0,05); CCL2 (0,77 and 0,5); CXCL8 (1 and 0,64); VEGF: (1 and 0,66); PTX-3: (0,55 and 0,90). ROC cut-off values: IL-1 (0,81 pg/ml), IL-6 (5,86 pg/ml), IL-10 (4,14 pg/ml), TNFa (49,72 pg/ml), CCL2 (38,03 pg/ml), CXCL8 (7,01 pg/ml), VEGF (39,03 pg/ml), PTX3 (13,68 pg/ml). (PDF) [file pone.0148186.s001.pdf]
